# Iron for Africa—Report of an Expert Workshop

**DOI:** 10.3390/nu9060576

**Published:** 2017-06-05

**Authors:** Martin N. Mwangi, Kamija S. Phiri, Abdelhak Abkari, Mory Gbané, Raphaelle Bourdet-Sicard, Véronique Azaïs Braesco, Michael B. Zimmermann, Andrew M. Prentice

**Affiliations:** 1 Division of Human Nutrition, Wageningen University and Research, Wageningen 6700 AA, The Netherlands; mart.mwangi@gmail.com; 2 Nutrition and Health Department, School of Public Health and Community Development, Maseno University, Maseno, Kenya; 3 Public Health Department, College of Medicine, University of Malawi, Private Bag 360, Blantyre 8, Malawi; kamijaphiri@gmail.co; 4 Hassan II University, Faculty of medicine, CHU Ibn Rochd, Casablanca, Morocco; abkari60@gmail.com; 5 National Public Health Institute (INSP) and Nutrition Society of Côte d’Ivoire (SIN), Adjamé BPV 47, Abidjan 01, Côte d’Ivoire; morydoc@gmail.com; 6 Danone Nutricia Research, route de la Vauve, Palaiseau 91120, France; Raphaelle.BOURDET-SICARD@danone.com; 7 VAB-nutrition, rue Claude Danziger, Clermont-Ferrand 63100, France; 8 Institute of Food, Nutrition and Health, Swiss Federal Institute of Technology (ETH) Schmelzbergstrasse, Zurich 78092, Switzerland; michael.zimmermann@hest.ethz.ch; 9 MRC Unit The Gambia, Atlantic Road, Fajara, The Gambia & MRC International Nutrition Group, London School of Hygiene & Tropical Medicine, Keppel St, London WC1E 7HT, UK; Andrew.Prentice@lshtm.ac.uk

**Keywords:** iron deficiency, fortification, benefit-risk ratio, anemia, malaria, biomarkers

## Abstract

Scientific experts from nine countries gathered to share their views and experience around iron interventions in Africa. Inappropriate eating habits, infections and parasitism are responsible for significant prevalence of iron deficiency, but reliable and country-comparable prevalence estimates are lacking: improvements in biomarkers and cut-offs values adapted to context of use are needed. Benefits of iron interventions on growth and development are indisputable and outweigh risks, which exist in populations with a high infectious burden. Indeed, pathogen growth may increase with enhanced available iron, calling for caution and preventive measures where malaria or other infections are prevalent. Most African countries programmatically fortify flour and supplement pregnant women, while iron deficiency in young children is rather addressed at individual level. Coverage and efficacy could improve through increased access for target populations, raised awareness and lower cost. More bioavailable iron forms, helping to decrease iron dose, or prebiotics, which both may lower risk of infections are attractive opportunities for Africa. Fortifying specific food products could be a relevant route, adapted to local context and needs of population groups while providing education and training. More globally, partnerships involving various stakeholders are encouraged, that could tackle all aspects of the issue.

## 1. Introduction

A one-day workshop was convened in Marrakesh (Morocco) in October 2016 to discuss issues around iron interventions in Africa, with a focus on iron fortification. Iron deficiency (ID) is the most common nutritional disorder in the world and estimates have attributed about half the cases of anemia worldwide to iron deficiency [1]. Although the worldwide prevalence of anemia has slightly decreased in the past 20 years, the situation still raises important concerns in Africa, especially in the Central and Western parts of the continent [2]. 

Experts from nine African countries (Algeria, Côte d’Ivoire, Egypt, Ghana, Kenya, Malawi, Morocco, South Africa and The Gambia) from academia or governmental bodies contributed their scientific, clinical or public health expertise and their field experience to share knowledge and visions regarding the current situation and the most appropriate ways to address the specificities of ID in Africa. 

The objective of the workshop was to provide a landscape analysis of the needs and concerns facing African countries and to exchange knowledge on best practices that are currently in place or could be initiated in the different areas. The workshop covered three topics:

(i) the prevalence and assessment of ID;
(ii) the benefit/harm ratio of iron; and
(iii) the national strategies to fight ID and their impact.

This report summarizes the content of the presentations given by the authors and reflects the information, opinions and statements given by all the participants; it does not aim to be exhaustive. In this report, the words “anemia” and “iron deficiency” encompass anemia from all causes and iron deficiency from all causes, except when the wording “iron deficiency anemia” is used. 

## 2. Iron Deficiency in Africa: Prevalence, Causes and Diagnosis Tools

The prevalence of anemia (from all etiologies) and of ID in the represented African countries is displayed in Table 1, as provided in the presentations and complemented by the participants throughout the workshop. Prof. Abkari presented anemia and ID prevalence data and its causes in Morocco. Further data about anemia prevalence in African young children and pregnant women can be found in WHO-based documents [2] and in the African Demographic and Health Surveys [3], and a selection of prevalence data is presented in Table 1. 

Although the diverse methodologies of survey prevent direct comparisons amongst countries, and despite numerous missing values, the prevalence of anemia from all causes and of iron deficiency (with or without anemia) appear elevated across the African continent, with South Africa consistently displaying the lowest figures. Anemia is widespread elsewhere, and affects more than 70% of young children and more than 45% of women in countries such as Côte d’Ivoire, The Gambia or Malawi. Iron deficiency often concerns more than half of young children except in Kenya, but less so in women, except in Egypt, as prevalence remains below 18%.

Low dietary intakes of iron are a major cause of ID in all represented countries. Consumption of animal products, containing high amounts of bioavailable heme iron, is limited by cost and availability issues. In addition, African diets are usually not rich in vitamin C, which enhances iron absorption, and they often contain chelators which bind iron in the digestive tract and limit its absorption [19]. In Africa, foods from plant sources, such as cereal- or legume-based flours, are often rich in phytates, and many common foods or beverages may contain iron-binding phenolics. This is the case in Morocco where tea, rich in polyphenols, is the national drink consumed throughout the day [20], including by very young infants [21]. Although tea is consumed in other African countries, it is at a lower rate and its contributing role in ID is thought to be less than in Morocco.

The nature of foods given to the child during complementary feeding appeared key to all participants. Indeed, iron stores at birth are usually adequate, because of maternal transfer; they drastically decrease between the 2nd and the 5th month of life. Complementary feeding practices are often detrimental to iron stores, especially when the child is switched early to iron-poor cow’s milk, or suddenly given food from the shared family dish. In a growing number of situations, children may be introduced very early to unhealthy items (such as soft drinks) which do not help iron supplies and may pose threats to the acquisition of healthy eating habits. 

Besides diet, other reasons contribute to explain the high level of ID and anemia in African young children and women. Firstly, these age groups have increased requirements, linked to growth, pregnancy, lactation or menstrual blood losses. Secondly, idiopathic malabsorption, such as those due to celiac disease, can remain undiagnosed in many African populations, although they reach significant levels [22]. Thirdly, infections and parasitic infestations, leading to chronic blood losses, thus iron losses, are frequent in many African countries, although infectious agents may differ [23,24]. Malaria is endemic in many areas of Sub-Saharan Africa, and many populations are also suffering from other parasites and chronic infections, e.g., cytomegalovirus or hepatitis. North African countries do not experience malaria and are less exposed to parasites [25], but they experience a high and possibly growing prevalence of *Helicobacter pylori*, especially in Algeria, but also in Egypt and Morocco [26]. A recent meta-analysis concluded on a plausible link between *Helicobacter pylori* infection and ID [27] but studies that have measured iron absorption in individuals with *Helicobacter pylori* infection have produced mixed results [28,29,30,31], and the role of *Helicobacter pylori* in the etiology of ID in Africa remains uncertain.

The African continent as a whole thus concentrates a high number of factors that increase the risk of ID. However, regions and even countries within the same African region, differ in the pattern of risk factors; for example, malaria is more prevalent in Sub-Saharan Africa than in the Maghreb; within Northern Africa, dietary habits are different between Morocco, where iron-chelating tea is highly consumed, and the neighbor Algeria where this drink is much less popular. These examples led the participants in the workshop to conclude that strategies to fight ID must consider regional and national specificities. 

Iron deficiency is most commonly assessed using hemoglobin (Hb) concentration. Limitations with this method include lack of specificity (all causes of anemia affect Hb concentration) and sensitivity as a drop in hemoglobin is a late manifestation of ID. ID can also be estimated using blood ferritin level, which assesses the size of body iron stores. However, ferritin is an acute phase reactant and it is elevated during infection or inflammation thus confounding the interpretation. Other biomarkers of iron include serum iron, serum transferrin, total iron binding capacity (TIBC) and unsaturated iron binding capacity, transferrin saturation, transferrin-ferritin index (TfR-F), soluble transferrin receptor (sTfR), zinc protoporphyrin, mean cell volume or mean cell hemoglobin concentration (MCHC). A summary of their meaning, practicalities, advantages and disadvantages is available elsewhere [32]. 

In addition, the setting of appropriate cut-off values for each one of these markers to identify ID in a consistent and comparable way is still a scientific challenge, as was illustrated during the workshop by Prof Phiri, who compared a wide range of ID markers in 381 severely anemic Malawian children (mean age: 20 months) [33]. The prevalence of ID according to each marker was assessed using internationally accepted WHO cut-off values and compared to the prevalence of ID estimated via bone marrow iron, considered as the “gold standard” measure, in spite of its invasiveness and subjectivity [34]. Sixty percent of children had malaria parasites and CRP was raised in 89% of them, indicating that inflammation was present in most children.

Box 1 **Hepcidin has been mentioned across the various sessions of the workshop.**

- This peptide acts as *the master regulator of iron* (^9^: it controls its dietary absorption, storage, and tissue distribution.
- Hepcidin integrates signals from iron in serum, liver and bone marrow and from inflammation; it could thus act as a *biomarker reflecting iron status*, but its effective use as such still needs more research.
- Hepcidin prevents iron absorption in inflammatory contexts and may blunt the efficacy of iron interventions in such contexts.

Depending upon the different markers used, ID was detected from 1% (through TIBC) to 97.5% (through serum ferritin) of children, demonstrating a clear lack of consistency. When specificity and sensitivity were computed, a reasonably good performance was found for four markers: ferritin, sTfR, TfR-F index and MCHC. New cut off values were proposed for these markers, sometimes very different from conventional ones; TfR-F index and MCHC were thought to be the preferable tools to assess ID in such a population of severely anemic and infected young children. 

Many of the workshop participants confirmed challenges using currently recommended cut-off values and were keen to have more robust standardized guidelines. Similar studies would be welcomed in populations of different countries, ages and infectious or parasitic status, in order to know which markers and cut-offs would be the most appropriate. The need for a simple and reliable biomarker was strongly expressed, and several participants acknowledge that hepcidin, a protein which plays a central role in iron regulation, including absorption, recycling, and tissue distribution (see Box 1 and [35]) could play this role. Indeed, there was a consensus that detecting individuals and populations who are really suffering from ID are of utmost importance: not only to clarify knowledge and statistics about prevalence rates, but most importantly to identify the right target populations for iron interventions. 

However, concerns were raised that the multiplication of markers and cut-off values would not ease the comparison across countries or the standardization of methods and would add even more complexity for policy makers. It was agreed that WHO guidelines should be followed for now, at least until evidence becomes available from current initiatives, such as the BRINDA project which aim at improving the interpretation of ID biomarkers in various contexts of infection burden; several methods are being proposed, including a regression model taking inflammation into account, instead of using ferritin cut-off value [36]. However, until such models are validated and accepted, serum ferritin remains the preferred marker, except where inflammation is prevalent, in which case the use of soluble transferrin receptor may be more appropriate [32].

Box 2 **Highlights of session about prevalence and diagnosis of iron deficiency (ID).**

- Precise, reliable and comparable data on the prevalence of ID are lacking in many countries and population groups. Among other factors, this is due to biomarkers being frequently biased by inflammation.
- ID is a worrying reality in young children and women, which does not seem to be currently decreasing.
- ID-associated factors vary according to regions and countries, but inappropriate eating habits, and high burdens of infections and parasitic diseases are critical.

**Research needs**

- Initiate large epidemiological surveys on representative populations and with appropriate ID biomarkers including inflammatory markers.
- Focus on post-partum anemia and ID, which are under-studied.
- Improve ID biomarkers and their cut-offs and set clear interpretations according to context of use.

## 3. Benefits and Risks of Iron Interventions 

As stated by one of the presenters during the workshop, “iron is probably the most widely used therapeutic in the world, but without a real test of its efficacy/risk balance”. This paradox is raising a growing concern, particularly in settings with a high infection burden, which is addressed by several agencies or scientific bodies, such as the IUNS (International Union of Nutrition Societies) [37] so far without definitive and operational answers. 

As highlighted by Dr. Mwangi, an appropriate iron status is essential to enable each child to become a “five star” adult, fully developed both physically and cognitively. Iron is important at each developmental stage, from the fetal life, to infancy, childhood, then teen and adult ages and WHO has developed guidelines for iron supplementation in most of these groups [38,39,40]. 

One out of 10 maternal deaths can be attributed to iron deficiency anemia [41,42]. According to the conclusion of a 2015 meta-analysis, preventive iron supplementation reduced maternal anemia at term by 70%, but data about maternal death, coming from two studies only, were inconclusive [43]. In an additional recent intervention trial in Kenya, anemia at birth affected 22% of women after iron supplementation vs. 50% of non-iron-supplemented women [44]. Ensuring an appropriate iron status post-partum is often neglected, while it is likely that low iron status are highly prevalent in this group, as 50–80% of women are anemic in low income countries [45]. Postpartum anemia is associated with an impaired quality of life, reduced cognitive abilities, emotional instability, and depression, which alter the interactions with the baby, with potentially negative impact on infant behavior and development [46]. 

Iron deficiency is rarely encountered in newborns, who obtain their iron stores via placental transfer from the mother’s iron stores. Optimal maternal iron status is beneficial for the newborn as it increases the transfer of iron to the newborn and may increase birth weight. In the above quoted study, iron-supplementation of ID mothers led to a birth weight being increased by 234 g, compared to 149 g in non-ID mothers and fewer than 17 women needed to be supplemented to avoid one case of low birth weight. Infant iron stores may become depleted starting ages 4–6 months, as stores transferred during pregnancy are depleted at a time when requirements are high because of rapid growth and erythropoietic needs. Iron deficiency at this period and up to school age may irreversibly affect cognitive development and physical growth and provision of iron has shown positive effects in improving global cognitive scores, intelligence quotient and measures of attention and concentration. Iron supplementation also improved age-adjusted height and weight among all children; the strength of the conclusions, however, varies according to studies, ages and outcomes [47,48]. 

Risks associated to iron interventions used to be limited to iron overload, with potential risks of tissue damage and oxidative stress [49]. These have long been known to occur in conjunction with genetic defects, such as beta-thalassemia or hereditary hemochromatosis, and/or in conditions which require regular blood transfusions [50]. From the early 2000s, concerns were raised about the effect of iron supplementation on increased susceptibility to infection, based on a potentially enhanced growth of pathogens from available iron. In 2006, the results of the Pemba study demonstrated that, in children living in malaria-endemic settings, iron and folate supplementation increased all-cause mortality and hospital admissions vs. a placebo control [51]. More concern is now given to the infectious context when implementing iron intervention, and recent systematic reviews have concluded that, overall, iron supplementation does not cause an excess of clinical malaria in children when proper malaria prevention and treatment is implemented [52], which is however often not possible in low-resource settings. Other infections could be affected by iron supplementation, as demonstrated by findings of an increased respiratory morbidity in iron-supplemented South African children [53], of diarrhea [54] or of increased infectious outbreaks in HIV positive Malawian children [55]. In a meta-analysis of trials in children aged 6–24 months, diarrhea, vomiting and fever were more prevalent in children receiving iron [1]. 

As early as 2002, a small, but significant increase in diarrhea had been reported in a systematic review of the effect of iron supplementation on incidence of infectious illness in children [56]. The mechanism of this effect may be explained by the fact that iron is a growth-limiting nutrient for many pathogenic gut bacteria, a topic developed during the workshop by Prof. Zimmermann. Indeed, pathogenic strains compete for unabsorbed dietary iron in the colon [57], whereas bifidobacteria and lactobacilli, which exert colonization resistance versus pathogens require little or no iron [58]. Clinical demonstration of these microbiological considerations has been provided in a six-month trial on anemic Ivorian schoolchildren receiving a biscuit containing 20 mg electrolytic iron four times a week or a placebo. Iron fortification caused a five-fold increase in enterobacteria, a five-fold decrease in lactobacilli and a five-fold increase in fecal calprotectin, an inflammatory marker [59]. These findings were confirmed in a controlled trial of multi-nutrient powder (MNP) in home fortification, in a younger population of Kenyan 6-month old children, whose microbiota was initially composed of 63% bifidobacteria but was also highly contaminated with pathogens. Iron supplementation significantly increased both the ratio of enterobacteria to bifidobacteria and enterobacteria to lactobacilli, increased inflammation and increased pathogenic *E. coli*. Diarrhea occurred in nearly 30% of children receiving iron, vs. in 8% of those who did not (non-significant difference) [60]. These findings raise safety concerns for African infants for whom increased dietary iron intake may enhance susceptibility to diarrhea and possibly, bacteremia and sepsis.

In adults, and especially in pregnant or post-partum women, it would be plausible that iron supplementation could pose a potential risk of higher infections, for the same biological reasons as in children. It has indeed been observed that iron deficiency is associated with a reduced prevalence and density of *Plasmodium* parasites in placental blood; it is concerning that potential effects of iron interventions in increasing malaria could be more pronounced in pregnancy, when iron absorption is higher. However, no evidence of an enhanced *Plasmodium* infection has been so far demonstrated, including in a recent study on Kenyan women supplemented during pregnancy [61]. It should be pointed out that these women received preventive treatment for malaria from regular health services during the study. 

During the lively discussion following both presentations in this session, the overall feeling was that iron interventions for vulnerable populations such as infants and young women are still needed and that their benefits generally outweigh their risks. However, there was consensus that such interventions should be carried out with caution, especially in children with infectious risk. Safety of iron interventions can be further promoted by an appropriate control of worm infections, malaria and other infectious risks. Recently reported improvements in malaria control in Africa may raise some hope in this regards [62]. 

Discussion also focused on individualization of iron interventions, which should probably be preferred to population-wide programs, in order to target those who need and who are not at risk of unfavorable outcomes of iron intake. Individual decisions, taken at the clinic, by practitioner would enable targeted and probably safer approaches. Indeed, even though malaria is not present everywhere in Africa, many other conditions may exist that interfere with the benefit/risk ratio of iron interventions, such as high prevalence of HIV, such as in Malawi [55]. Other concerns include a high overall infectious risk in young children, usually not monitored or controlled, and the frequent inappropriate use of antibiotics, additionally perturbing the gut microbiota. In addition, the potential interaction of iron with other micronutrients, such as vitamin A, riboflavin and iodine, should be considered.

Box 3 **Highlights of session about benefits and risks of iron interventions.**

- *Clear benefits* regarding pregnancy, growth and development:
  - Decreased maternal anemia
  - Increased infant’s birth weight
  - Improved cognitive capacity of children under 2 years
- *Potential risks* may in high infectious context:
  - Enhanced pathogen growth (increased availability of iron in blood and unabsorbed iron in the gut).
  - Increased infectious risk in the absence of monitoring and treatment programs.
- However, *benefits outweigh risks*, in particular when infection can be monitored and controlled.
- *Caution should* remain, especially when infants and children are concerned and, if possible, interventions should be targeted to iron-deficient subjects.

**Research needs**

- Better assessments of the impact of iron intervention on growth outcomes and cognition in younger children.
- Exploration of the harms of high/excessive iron status and associated outcomes, especially in pregnancy.
- Investigations on the impact of iron on gut microbiota in various populations (e.g., pregnant women).

## 4. Strategies for Iron Intervention

Because ID has long been recognized as a major global public health problem, international agencies, governments of concerned countries and non-governmental organizations have been working on potential solutions to control ID, as summarized by Prof. Prentice. These include:

- Increasing the dietary supply of iron-rich foods, among which animal-sourced ones offer highly bioavailable iron. This ideal solution is often difficult to implement for economic and practical reasons.
- Delaying cord clamping at birth is a simple but efficient means to increase the infant’s body iron stores, which could be developed through appropriate education of health care professionals.
- Fortifying some foods within the usual diet can be done centrally on the whole supply of staple foods such as flour, without targeting population groups or individuals. Home fortification with MNPs or industrially fortified processed foods (e.g., biscuits, cereals, infant formulae) may allow some personalization and thus a better adaptation to individual’s needs.
- Supplementing vulnerable groups is recommended by WHO for populations, living in settings where anemia prevalence is over 40%, including menstruating women [38], post-partum women [39] and children above six months [40]. According to WHO guidelines, supplementation is today most often recommended on a daily basis.

Strategies currently implemented at the national level in African countries are diverse. Dr. M. Gbané detailed those existing in Côte d’Ivoire and participants shared their experience about practices in their respective countries. 

Fortification of staple foods (wheat and/or maize flour) exists in all represented countries but Algeria [63]. Iron compounds commonly used are either electrolytic iron (35 ppm in South Africa, or 45 ppm in Morocco), water soluble forms (60 ppm ferrous fumarate or sulfate in Côte d’Ivoire, Egypt, The Gambia and Ghana), or sodium-Ethylene Diamine Tetraacetic Acid chelated (EDTA) forms (5 to 50 ppm) in Kenya or Malawi [63]. The coverage is however often low, such as in Morocco, where only 35% of bakers using fortified flours in urban areas and in Côte d’Ivoire where the country coverage of fortified flour does not exceed 14%, due to political crises. 

Point of use fortification, using MicroNutrient Powders (MNP), is a more targeted approach, implemented in a few countries, such as Ghana or Kenya [64], but with a low adherence of populations after the first few months. Some iron-fortified infant and young children foods (formulae, growing up milk, cereals, etc.) exist in several countries (Algeria, Côte d’Ivoire, Egypt, etc.), but their use is usually not widespread. 

Policies for targeted supplementation of women or young children vary from one country to another. Malawi has no country-wide program, whereas Morocco, Kenya and Côte d’Ivoire have implemented intermittent supplementation for women, but none for children. Algeria targets women and at-risk newborns (premature, low birth weight and twins). The Gambia recommends daily supplementation of all pregnant women.

Other public health policy measures are taken by countries in order to improve the efficacy of iron interventions via raising awareness and knowledge of health care professionals and populations. In Algeria, for example, the AAPNEM (Association Algérienne pour la Nutrition de l’Enfant et de la Mère) develops educational programs for mothers, but also for health care professionals and social workers. In Egypt, an awareness campaign is planned in 2017. 

The opinion of the participants was that none of these strategies have been completely effective, and trends from surveys on Hb levels and anemia prevalence from 1990 to 2010 appear to confirm this feeling; indeed, in several African countries, these figures have not improved and may even have worsened in a few cases [2]. Discussion showed that this could be due to a myriad of different reasons, more or less important according to countries and situations, among which participants identified:

- The difficulty of identifying and then reaching the target population groups and individuals.
- The lack of awareness, knowledge and understanding (especially regarding for home fortification) of caregivers, but also health professionals.
- The poor adherence to programs or prescriptions.
- The poor availability of iron-containing supplies (supplement, fortified staple foods, and fortified products), which may be missing at point of supply or purchase.
- The cost: Even for government-funded supplementation programs, which is not always the case, the subject should often pay a part of the cost. Iron-fortified products are often too expensive for the populations who would need them.
- The low bioavailability of some iron forms. Several countries, such as Morocco, are currently considering a change to the more bioavailable NaFeEDTA in their mandatory fortification programs.
- The infectious context: Infants and young children may be especially vulnerable to infection, both because they are exposed to pathogens and because their immune system is still immature. In addition, infection-induced hepcidin secretion limits iron absorption in contexts where the hygiene level is low [35].

The discussion also addressed the actions aimed at minimizing the risks linked to iron interventions. A first requirement should be to target only populations and individuals who really need iron. Screening for ID is thus seen as a mandatory step before implementing interventions, with a marked preference for not relying only on anemia prevalence. The second requirement should be a better control of the infectious and inflammatory risk in targeted populations: deworming, malaria prevention (mosquito nests) and treatment and increased water supply are needed to improve hygiene and sanitation and reduce pathogens, as promoted by UNICEF in its WASH (Water, Sanitation and Hygiene) programs.

Education also appeared to all participants as a powerful lever to fight ID. Hygiene education and nutrition education are equally important in raising women’s and mothers’ awareness and understanding, enhancing their commitment and adoption of good practices. Education should stress the importance of iron for them and their children and promote exclusive breast feeding during the first months of life, appropriate complementary feeding and appropriate dietary diversification. Providing training to health care professionals is seen as critical to keep them aware of the risk of iron intervention in infected children and the need to screen for ID with appropriate markers and cut-offs. Professionals involved around delivery should know better about the importance of delayed cord clamping. Finally, health-care professionals should also be sensitized to the need of considering that, beyond young children, school children, post-partum women and child-bearing age women may also be at risk of ID.

Another approach to cope with potential harms of iron interventions is to reduce the iron dose as low as possible by maximizing absorption. The amount of supplemental iron provided daily can be significantly reduced if efforts are made to increase its bioavailability by using well-absorbed forms of iron and enhancers of iron absorption. In a study on South African school-aged children with a low iron status, Prof Zimmermann’s team demonstrated that providing only 2.5 mg of iron as NaFeEDTA together with ascorbic acid and exogenous phytase active at gut pH decreased the prevalence of ID by more than 75% vs. 35% in the control group [65]. Indeed, a lower iron dose would result in less unabsorbed iron entering the distal gut and would be expected to decrease the risk of iron-induced pathogen growth in the gut, and of associated infections. Another potential approach could be to co-fortify prebiotic components, such as galacto-oligosaccharides (GOS) with iron in MNPs, in order to support bifidobacteria in children’s microbiota, thus maintaining colonization resistance and preventing or decreasing pathogen growth following iron supplementation. This has been tested by Prof. Zimmermann’s group in 155 Kenyan infants aged 4–7 months, in a four-month long trial during which children were randomized into three groups, which received either no iron or 5 mg iron added or not to 7.5 g of GOS. ID prevalence decreased significantly in both iron-supplemented groups vs. in the no iron group. Furthermore, microbiota of children receiving iron + GOS had a more favorable profile (more *Lactobacilli*, more *Bifidobacterium longum*, less pathogenic *E. coli*, lower ratio of *Enterobacteriaceae* over *Lactobacilli* and *Bifidobacteria*) when compared to children receiving iron alone. This was associated with a lower rate of upper respiratory infections and diarrhea in children treated with iron + GOS, compared to both other groups. These data, still unpublished, were seen as extremely promising by the audience, although deserving confirmation in other studies and in other age ranges.

Box 4 **Highlights of session about iron intervention strategies.**

- Many African countries have governmental programs to fortify flour and to supplement pregnant women; supplementation of children is not systematic.
- Coverage and efficacy of these actions are variable, but usually far from optimal, for many reasons (difficulty to reach the target population, cost and iron bioavailability of supplement/fortified foods, lack of awareness and compliance, etc.).
- The risk of increased infections, especially in young children, acts as a bottleneck in areas with high infectious disease burden.
- Improving availability of iron (chelated forms, absorption enhancers, etc.) would help to lower iron dose, thus to decrease harms, while keeping a similar efficacy.
- Using prebiotics together with iron could lower risk of enteropathogen growth and infections in infants.

**Research needs**

- Demonstrate that increased clean water availability, washing practices and overall hygiene increase the safety and efficacy of iron intervention in young children.
- Confirm and extend studies about prebiotics and determine which one(s) would be best adapted to an African context and to various ages and populations.

## 5. Fortified Food Products: Which Products for Which Target Group?

The final discussion focused on practical recommendations that could be given regarding iron fortification in Africa. 

Regarding targets of such fortified food products, there was a consensus that children aged six months to three years are of primary concern. As this target is also the vulnerable to infections, it implies a careful monitoring of risks associated to iron fortification: knowledgeable parties should be involved, which means that education and training should be implemented together with fortification. Screening for ID and for infection should also be performed. Besides young children, other targets should be considered, namely school aged children, adolescents and women in the periconceptional period, as well as pregnant and post-partum women. 

Preferred iron forms could include NaFeEDTA, or ferrous fumarate or sulfate, because of their high bioavailability, along with ascorbic acid as an absorption enhancer; however, because it is sensitive to heat and oxidation, ascorbic acid requires some technical expertise to remain intact in food products. Iron bisglycinate is a well-absorbed form of iron, although potentially costly; other iron forms such as iron hydroxide adipate tartrate (IHAT) that are currently under investigation in Africa deserve future attention. The iron dose in a fortified food product should provide at a level equivalent to at least 20% of the dietary requirement, but is not intended to cover the entire daily iron supply: this low dose should lower the harms associated with iron.

The food vehicle or matrix should be adapted to target- and country-relevant dietary habits, and should be available at an affordable cost. It should also be a food appreciated by the target groups (for children, cereal or biscuit, or porridge drinks for women, for example). Including prebiotics in iron-fortified products appears as a very promising route, especially in low hygiene environments, but this needs further research, both to confirm efficacy and to address practicalities (type of prebiotic, dose, technology and costs). It was clearly stressed that iron fortified food products should have a favorable nutritional profile in addition to iron content and should not introduce inappropriate dietary habits, i.e., should not favor overconsumption or unbalanced diets. Programs should ensure that the fortified products are eaten by those who need them most. 

## 6. Conclusions 

This workshop was an opportunity for experts from various backgrounds and countries to share their experience and questions around ID, which remains a public health problem on the African continent. It was clear to all participants that Africa is changing quickly and that there are reasons to be optimistic about its improving capacity to reduce ID. As a country/continent develops economically and gets better infrastructure, namely, in the sanitation, education and health domains, many infectious diseases will become easier to decrease or eliminate. The challenge would be to find the optimal route that avoids the “double nutrition burden” (i.e., coexistence of deficiencies and obesity), which means choosing smart interventions that fight disease and micronutrient deficiencies without predisposing the population to metabolic diseases.

Among the routes offered to Africa to better and more efficiently address ID, the opportunities offered by developing partnerships among various stakeholders should be better exploited. These could include projects involving several or all entities among governments, international organizations and non-governmental associations, but also industries, including food industries. By joining different forces and expertise the issue of ID will be more efficiently tackled, in all its dimensions. 

## Figures and Tables

**Table 1:** Prevalence (%) of anemia from all causes (A) and of iron deficiency (ID) in selected African countries.

Age Group	Algeria		Egypt		Côte d’Ivoire		The Gambia		Ghana		Kenya		Malawi [4]		Morocco		South Africa [5]	
	A *	ID	A	ID	A	ID	A	ID	A	ID	A	ID	A	ID	A	ID	A	ID
Children below 5	64 [6]	51 [6]	27 [7]	64 [8]	75 [9]	51 [10]	73 [11]	NA	66 [12]	NA	46 [2]	21 [13]	73	NA	30 [14]	NA	10.5	11
Child-bearing age women	NA	NA	39 [4]	51 [15]	54 [9]	17 [10]	60 [11]	NA	42 [12]	16 [16]	NA	NA	46	NA	33 [17]	NA	23	15

* A: anemia; ID: Iron deficiency; NA: data not available. Data come from the most recent available sources. Data from representative surveys have been preferred (see references for date and scope of surveys). Anemia was identified by Hb blood levels below 110 mg/dL in children and pregnant women and 120 mg/dL in non-pregnant women [18].

## Abbreviations

Hb	Hemoglobin
GOS	Galacto-OligoSaccharides
ID	Iron Defiency
IUNS	International Union of Nutrition Societies
MCHC	Mean Cell Hemoglobin Concentration
MNP	MicroNutrient Powders
sTfR	Soluble Transferrin Receptor
TfR-F	Transferrin-ferritin Index
TIBC	Total Iron Binding Capacity
